# Increased Expression of Simple Ganglioside Species GM2 and GM3 Detected by MALDI Imaging Mass Spectrometry in a Combined Rat Model of Aβ Toxicity and Stroke

**DOI:** 10.1371/journal.pone.0130364

**Published:** 2015-06-18

**Authors:** Sarah Caughlin, Jeffrey D. Hepburn, Dae Hee Park, Kristina Jurcic, Ken K.-C. Yeung, David F. Cechetto, Shawn N. Whitehead

**Affiliations:** 1 Dept. Anatomy and Cell Biology, Western University, London, ON, N6A 5C1, Canada; 2 Dept. Chemistry and Dept. Biochemistry, Western University, London, ON, N6A 5C1, Canada; 3 Dept. Clinical Neurological Sciences, London Health Sciences Centre, University of Western Ontario, London, ON, N6A 5A5, Canada; Universidad de Castilla-La Mancha, SPAIN

## Abstract

The aging brain is often characterized by the presence of multiple comorbidities resulting in synergistic damaging effects in the brain as demonstrated through the interaction of Alzheimer’s disease (AD) and stroke. Gangliosides, a family of membrane lipids enriched in the central nervous system, may have a mechanistic role in mediating the brain’s response to injury as their expression is altered in a number of disease and injury states. Matrix-Assisted Laser Desorption Ionization (MALDI) Imaging Mass Spectrometry (IMS) was used to study the expression of A-series ganglioside species GD1a, GM1, GM2, and GM3 to determine alteration of their expression profiles in the presence of beta-amyloid (Aβ) toxicity in addition to ischemic injury. To model a stroke, rats received a unilateral striatal injection of endothelin-1 (ET-1) (stroke alone group). To model Aβ toxicity, rats received intracerebralventricular (icv) injections of the toxic 25-35 fragment of the Aβ peptide (Aβ alone group). To model the combination of Aβ toxicity with stroke, rats received both the unilateral ET-1 injection and the bilateral icv injections of Aβ₂₅₋₃₅ (combined Aβ/ET-1 group). By 3 d, a significant increase in the simple ganglioside species GM2 was observed in the ischemic brain region of rats who received a stroke (ET-1), with or without Aβ. By 21 d, GM2 levels only remained elevated in the combined Aβ/ET-1 group. GM3 levels however demonstrated a different pattern of expression. By 3 d GM3 was elevated in the ischemic brain region only in the combined Aβ/ET-1 group. By 21 d, GM3 was elevated in the ischemic brain region in both stroke alone and Aβ/ET-1 groups. Overall, results indicate that the accumulation of simple ganglioside species GM2 and GM3 may be indicative of a mechanism of interaction between AD and stroke.

## Introduction

As we age, our brains become more vulnerable to diseases and injuries. Elderly patients often simultaneously experience two or more medical conditions which complicates the study of age-related neurodegenerative diseases such as Alzheimer’s disease (AD). When multiple conditions are present simultaneously (comorbidity), synergistic effects on pathology and cognitive outcomes can be observed as demonstrated in the case of AD and stroke. A key study which examined the incidence of dementia among a group of elderly nuns diagnosed with AD found that only 57% of those diagnosed with AD developed dementia, while 93% of those who had suffered small subcortical infarcts plus a pathological diagnosis of AD developed dementia [1]. AD and stroke comorbidity has also been observed in carriers of the APOE4 gene. Data from the Canadian Study of Health and Aging (CSHA) showed that prevalence of dementia was increased among those who had a history of stroke and were also APOE4 carriers [2]. Furthermore, another study demonstrated that APOE4 carriers with a history of stroke were five times more likely to develop dementia than APOE4 carriers without such a history [3]. Although the clinical evidence for the interaction between AD and stroke has been well documented, the mechanism(s) for this interaction remains unclear. A potential mediator for this interaction may lie within a family of cellular membrane lipids known as gangliosides.

**Fig 1:**
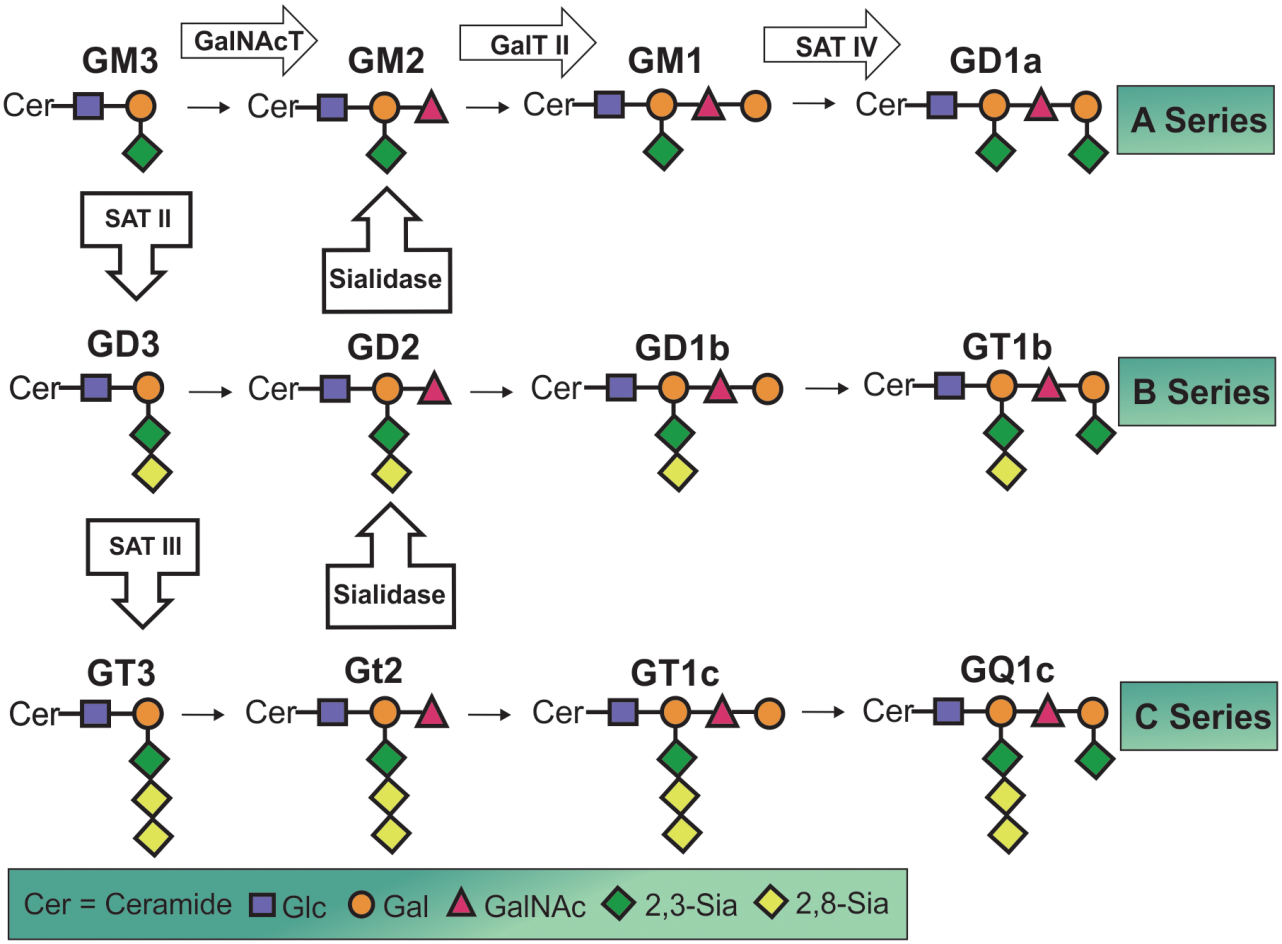
Chemical Structure and Metabolic Pathways Involved in Ganglioside Synthesis and Catabolism. A group of transferase enzymes add either a sugar unit (ex: along A-series pathway) or a sialic acid residue (ex: A to B series pathways) to synthesize different ganglioside species. A different set of enzymes breaks down these components to increase levels of simpler species thus a homeostatic level of each ganglioside species is maintained in a healthy organism.

Gangliosides are glycosphingolipids characterized by the presence of sialic acid residues. Being embedded within the plasma membrane, gangliosides perform a wide variety of biological functions by interacting with signalling molecules both within and outside of the cell. Some of these biological functions include cell signalling, proliferation, differentiation, embryogenesis, oncogenesis, neurodegeneration, and apoptosis [4,5]. Ganglioside metabolism is controlled by the activities of several enzymes, which can add or remove sialic acid residues and/or oligosaccharide units to form the various derivatives that make up the ganglioside molecules. A-series gangliosides are the first, structurally simplest group of gangliosides derived from the addition of a sialic acid residue to lactosylceramide and contain the most abundant ganglioside species in the mammalian central nervous system, GM1, as well as GD1a, GM2, and GM3 [6] (Fig 1). Every form of ganglioside is hypothesized to have unique functions within the cell and a normal homeostatic distribution of each species is maintained within healthy organisms [7–9]. Complex gangliosides GM1 and GD1a are more abundant in the brain than the simple species GM2 and GM3 and have been shown to be beneficial for recovery when exogenously administered in a number of *in vitro*, animal, and human disease and injury studies [10,11].

Little is known of the functions of ganglioside GM2 in the adult mammalian brain. However, GM2 gangliosidosis, a group of autosomal recessive disorders caused by dysfunction in enzyme metabolic pathways, such as Tay Sachs and Sandhoff’s disease, can lead to accumulation of GM2 in neuronal cells resulting in a broad spectrum of neurological disorders [12]. More recently, GM2 expression has been found to be elevated within the mouse brain at the injury site after a focal brain injury such as a traumatic brain injury or stroke [13,14]. GM3, an essential component of the plasma membrane and lipid rafts [4], is structurally the simplest member of the ganglioside family and acts as a metabolic precursor to the other more complex ganglioside species (Fig 1). As such, GM3 plays a crucial role in regulating the level of expression of all other ganglioside species in cells. Although GM3 is one of the main gangliosides expressed in extra-neural tissues of vertebrates, it is generally expressed only in very small quantities in the healthy adult mammalian brain [4]. Accumulation of GM3 in neural tissue has been shown to lead to toxic effects and apoptosis [4,15,16]. GM3 has been successfully used as a treatment for tumors due to its potent anti-angiogenic and apoptotic effects [17–19]. An increase in GM3 expression has also been observed in both human and animal models of AD [20], as well as in mouse models of stroke—reperfusion injury [13]. Finally, additional to the sugar chains, the functions of gangliosides may also be determined by ceramides. In particular, certain gangliosides differ only by the length of their sphingosine backbone, d18:1 and d20:1 (18 carbon and 20 carbon species), and have been suggested to have unique roles within the cell [13,14,21,22].

Labelling and visualizing gangliosides within tissue using immunohistochemistry (IHC) is a commonly used technique for identifying different species of gangliosides. However, the efficacy of antibody labelling on lipids in general is controversial and the availability of specific ganglioside antibodies for certain species can be very limited or non-existent [23]. Moreover, immunolabelling is also incapable of distinguishing between the d18:1 and d20:1 species as the ceramides are hidden within the cell membrane, inaccessible to antibodies for labelling. An alternative approach is the detection of gangliosides based on their molecular mass through spectrometry. Matrix-Assisted Laser Desorption/Ionization Imaging Mass Spectrometry (MALDI IMS) can detect ganglioside molecules directly within tissue sections without any kind of immunological labelling. The masses and intensities of all ganglioside ions are recorded simultaneously within an anatomical reference point.

Perturbations in the normal homeostatic expression of gangliosides have been observed in several models of neurodegeneration such as AD [24,25], Huntington’s disease [26], Parkinson’s disease [10], and prion diseases [27]; however, this has not yet been reported within the context of comorbidities. Previous work using the same animal model as this study, has demonstrated that a synergistic relationship exists between animal models of stroke and AD both pathologically and behaviourally [28,29], however the mechanism of this interaction remains unclear. This study utilizes both the novel MALDI IMS technique and, where possible, traditional IHC to examine changes in A-series ganglioside expression in rats who underwent either surgical striatal endothelin-1 (ET-1) injection (stroke alone group), bilateral intracerebralventricular (icv) injections of beta-amyloid (Aβ_25–35_) (Aβ alone group), or ET-1 with bilateral icv injections Aβ_25–35_ (combined Aβ/ET-1 group). The ET-1 subcortical stroke model was chosen as it produces subcortical lacunar type infarcts that resemble those typically seen in patients who, upon post-mortem examination, have been identified as having pathological correlates of both AD and stroke [1]. In addition, previous studies have demonstrated that targeted injections of ET-1 produced a transient focal ischemia-reperfusion injury in rats [30–32].

## Materials and Methods

All animal handling and surgical procedures were in accordance with guidelines of the Canadian Council on Animal Care and approved by the University of Western Ontario Animal Use Subcommittee. Adult male Wistar rats weighing 300–400 g (Charles River Laboratories, Quebec, Canada) were housed in pairs under standard conditions (12:12 light/dark cycle) and provided food and water *ad libitum*. Rats were randomly assigned to experimental surgical groups and were housed individually following surgery.

### Animal Models

Rats were weighed before being anaesthetized in a Harvard anesthesia box with 3% isoflurane in 2 L/min oxygen and placed in a Kopf stereotaxic apparatus. Using an operating microscope, an incision was made in the scalp and the skull was exposed. Bregma was used as a reference point for all injection sites: Injections were administered with a Hamilton glass syringe at a rate of 1 μL over 30 sec. The syringe was left in place for 3 min after each injection then slowly removed. Rat body temperature was maintained at 37°C on a heating pad during the surgical and recovery periods before being returned to a cage. To induce Aβ toxicity, rats received 15 μL bilateral icv injections of the toxic Aβ fragment, Aβ25–35 (50 nmol), at coordinates -0.8 mm (anterior/posterior), ±1.4 mm (medial/lateral) and -4.0 mm (dorsal/ventral). Aβ25–35 (Sigma-Aldrich Co., St. Louis, USA) was dissolved in saline and stored at -80°C in 30 μL aliquots and kept on dry ice prior to injection to prevent aggregation and allow better diffusion throughout the ventricles [12,30,31]. To induce stroke, rats received a 3 μL unilateral injection of the potent vasoconstrictor ET-1 (60 pmol) into the right striatum, at coordinates +0.5 mm (anterior/posterior), -3.0 mm (medial/lateral) and -5.0 mm (dorsal/ventral). ET-1 (Sigma-Aldrich Co., St. Louis, USA) was dissolved in sterile water and stored at -80°C in 8 μL aliquots and kept on dry ice prior to injection. To induce the comorbid Aβ/ET-1 model, rats received bilateral icv injections of Aβ25–35 followed by a unilateral ET-1 injection into the striatum at the same concentrations and coordinates as above. Sham surgery group animals underwent the same surgical procedure as the combined Aβ/ET-1 group rats but did not receive any chemical injections, only needle insertion to the appropriate brain regions. The mortality rate was slightly (but not statistically significantly) higher in ET-1 groups but was less than 10% of the total animals used and was attributed to the effects of the interaction between ET-1 and anesthesia.

### Euthanasia

For histochemical and IHC analysis, rats were euthanized at 3 or 21 d post-surgery via Euthanyl overdose (0.5 mL, i.p.). Animals were then perfused transcardially with 0.01 M phosphate buffered saline (PBS) (pH 7.4) followed by 4% paraformaldehyde (PFA) (pH 7.4). Brains were removed and kept in the same PFA solution for 24 hr, then cryoprotected in 30% sucrose until they were fully submerged. Brains were sectioned coronally on a Cryo3 Cryostat (TissueTek, Dublin, USA) into 35 μm thick sections and were stored in cryoprotectant at 4°C until used for immunohistochemistry. For MALDI IMS analysis, rats were euthanized via Euthanyl overdose (0.5 mL i.p.) at either 3 or 21 d post-surgery. Rats were decapitated and fresh whole brains were carefully removed and immediately frozen on dry ice. Frozen brains were stored at -80°C until used for MALDI IMS analysis.

### Immunohistochemistry (IHC)

Sections from each experimental group were processed simultaneously to reduce variability between groups. Sections were washed in 0.1 M PBS (3 x 15 min each), then quenched for 15 min in 1.5% hydrogen peroxide. Sections were washed in PBS (3 x 5 min each), then blocked in 1.5% bovine serum albumin (BSA) (Serological Research Institute, Richmond, USA) diluted in PBS with Triton-X (PBST) for 30 min. Sections were incubated with primary antibodies diluted in 1.5% BSA (PBST) for 48 hr at 4°C on a shaker. IHC procedure was similar for: mouse anti-rat OX-6 (1:1000, BD Biosciences, Mississauga, Canada), NeuN (1:1000, EMD Millipore, Billerica, USA), GFAP (1:1000, EMD Millipore, Billerica, USA) GM3 (1:500, Seikagaku Corporation, Tokyo, Japan), GM1 and GD1b (1:500, Ronald Schnaar, Johns Hopkins University, USA); however Triton-X was not used for GM3, GM1 or GD1b staining due to its membrane delocalizing effects [33]. Sections were then washed in PBS (3x 15 min each) then incubated with HRP-conjugated secondary antibodies diluted in 1.5% BSA (PBST) for 1 hr. HRP-conjugated (anti-mouse IgG) (1:200, Vector Laboratories, Burlington, Canada) and anti-rabbit IgG secondary antibodies (1:200, Vector Laboratories, Burlington, Canada) were used for the appropriate primary antibodies. Sections were washed in PBS (3 x 15 min each) then incubated in avidin-biotinylated complex (ABC) reagent (Vector Laboratories, Burlington, Canada) for 1 hr. Sections were then washed in PBS (3 x 5 min each) and developed in fresh 0.001% diaminobenzidine (DAB) (Vector Laboratories, Burlington, Canada) at 10 mg in 20 mL PBS with 300 μL 3% H2O2, Sigma for 5 min. Sections were washed in PBS (3 x 15 min each) then mounted onto VWR microscope slides coated in 0.3% gelatin, dehydrated, cleared with xylene and then cover-slipped with Depex (Fisher Scientific).

### Immunofluorescence

Free-floating sections were washed in PBS (3 x 10 min) then blocked in 1.5% BSA for 1 hr at room temperature. Sections were incubated with antibodies to GM3 [1:500] and either NeuN (1:1000, Millipore, Billerica, USA), GFAP (1:1000, Millipore, Billerica, USA) or IBA-1 for activated microglia/macrophages (1:1000, Santa Cruz Biotechnology Inc., Santa Cruz, USA) in 1.5% BSA for 48 hrs at 4°C. Sections were washed in PBS (3 x 10 min each) then incubated with FITC conjugated anti-mouse IgG (1:300, Santa Cruz Biotechnology Inc., Santa Cruz, USA) and TR-conjugated anti-rabbit IgG (1:300, Santa Cruz Biotechnology Inc., Santa Cruz, USA) for 1 hr at room temperature in the dark. Sections were washed in PBS (3 x 10 min) and mounted onto microscope glass slides and cover-slipped with Fluoroshield mounting media (Sigma-Aldrich, Toronto, Canada).

#### Fluoro Jade B

Sections were washed in PBS (6 x 10 min each) then mounted onto microscope slides with 0.3% gelatin and allowed to dry overnight. Slides were placed in 1% sodium hydroxide and in 80% ethanol for 5 min. Slides were then rehydrated in 95% ethanol for 3 min; 70% ethanol for 3 min; 50% ethanol for 2 min, and finally distilled water (3 x 1 min each). Slides were then incubated in 0.06% potassium permanganate (KMnO4) for 15 min on a shaker, in the dark. Slides were washed in distilled water (3 x 1 min each), then placed in fresh 0.0004% Fluoro Jade B (Millipore, Billerica, USA) in 0.1% acetic acid for 20 min on a shaker in the dark. Slides were washed in distilled water (3 x 1 min each) then allowed to dry in the fume hood before a xylene clearance step (1 min) and cover-slipped with Depex.

#### Microscopy Imaging

Images were taken on a Leica DC300 (Leica Microsystems, Concord, Canada) microscope camera. Analysis and quantification was carried out using Image J (Wayne Rasband, National Institute of Health, Bethesda, USA) by one observer who was blinded to the experimental groups.

### MALDI IMS

Fresh frozen rat brains were sliced on a CM 1850 cryostat and 10 μm coronal sections were taken from the striatum (stroke injury site) for MALDI IMS analysis. Sections were thaw mounted onto a MALDI target plate and dried in a desiccator for 10 min. An artist’s airbrush (Iwata HPBH, Japan) was used to apply 2 mL of 15 mg/mL 5-Chloro-3-mercaptobenzothiazol (CMBT) (Sigma-Aldrich, Toronto, Canada) matrix dissolved in a 4:4:1 mixture of chloroform:ethanol:water solvent, allowing each layer to dry completely before spraying the next layer thus minimizing delocalization of molecules within the tissue. Once the matrix was uniformly applied to the plate surface and dried, the plate was inserted into an AB Sciex MALDI 4700 TOF/TOF mass spectrometer (Applied Biosystems, Foster City, CA, USA). Prior to acquiring an image, the instrument was calibrated using five peptide standards (4700 Calibration Mixture, AB Sciex) at 50 ppm mass tolerance. Reflectron and negative ion modes were used for all image acquisitions. During the scanning process, a laser beam is directed across the selected tissue sections at 100 μm steps and a mass spectrum is collected for each x,y coordinate. The laser ablates the tissue and causes the desorbed molecules in the tissue to ionize. These ions travel down a flight tube where they separate based on their mass-to-charge (m/z) ratio to a detector which records this data and compiles it into both a mass spectrometry data plot and a molecular image. The instrument took approximately 2 hr for acquisition of each image.

**Fig 2:**
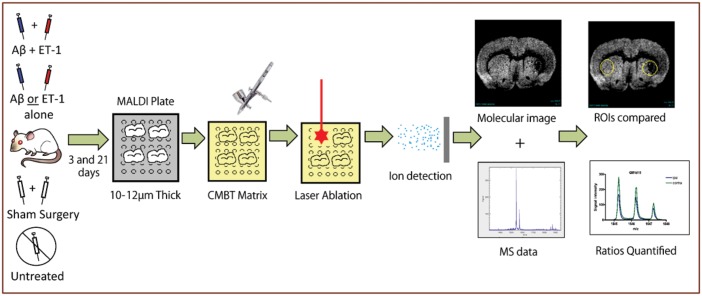
MALDI IMS Workflow. Following surgery, rats are euthanized via anesthetic overdose and decapitated. Fresh brains are isolated and sectioned at 10μm on a cryostat onto MALDI plates. Sections sprayed with CMBT matrix using an airbrush are processed on a MALDI mass spectrometer. Regions of interest (ROIs) are chosen based on adjacently sectioned and thionin stained brain sections. Stroke regions are identified and mass spectra are averaged from this infarct as well as from the corresponding non-injured striatum on the contralateral hemisphere. Mass spectra are generated from each ROI, and the areas under the curve for the species of interest is calculated. Quantified data are represented as the ratio of the area under the curve between the ipsilateral (stroke) and contralateral ROIs.

The region of interest (ROI) for this MALDI IMS study included both the core of the infarct and the periphery in order to assess general changes in ganglioside metabolism after stroke and Aβ toxicity within the site of stroke injury. Representative MALDI IMS images were selected for the ET-1 group and Aβ/ET-1 combined group for each species of ganglioside in order to better visualize the changes in expression. Representative IMS images from the Aβ alone and control groups were not included as there were no significant alterations in ganglioside expression in any of these groups. The location of stroke injury within the striatum is highlighted with a green arrow in the MALDI IMS images. Three day (d) and 21 d time points were examined for each A-series ganglioside. The appropriate m/z ratio for each ganglioside species was confirmed using the Lipidmaps database (www.lipidmaps.org). Data was collected and analyzed as previously described (Fig 2).

### Data Analysis

A single MALDI IMS image scan contains molecular information on the expression of each type of A-series ganglioside, the d18:1 and d20:1 species, as well as some unknown metabolic species. The molecular image can be used to locate a specific region of interest (ROI) such as the stroke region in the striatum where a mass spectrometry data plot is produced for that specific region for quantification. Multiple ROIs can be selected for comparison purposes. In this case, the injury site in one hemisphere was compared to the same location on the contralateral side of the brain. The expression of each A-series ganglioside was quantified in both ROIs and, after baseline subtraction, a ratio was calculated for the differences in expression in the injured and non-injured tissue. The ratios for multiple brain tissue scans in each group were compiled and a one way ANOVA was performed followed by a post-hoc Tukey’s multiple comparisons test to determine if there were any differences in expression between the surgical groups and the controls at both time points. A p-value of <0.05 was considered to be significant, n = 4 for each surgical group.

## Results

**Fig 3:**
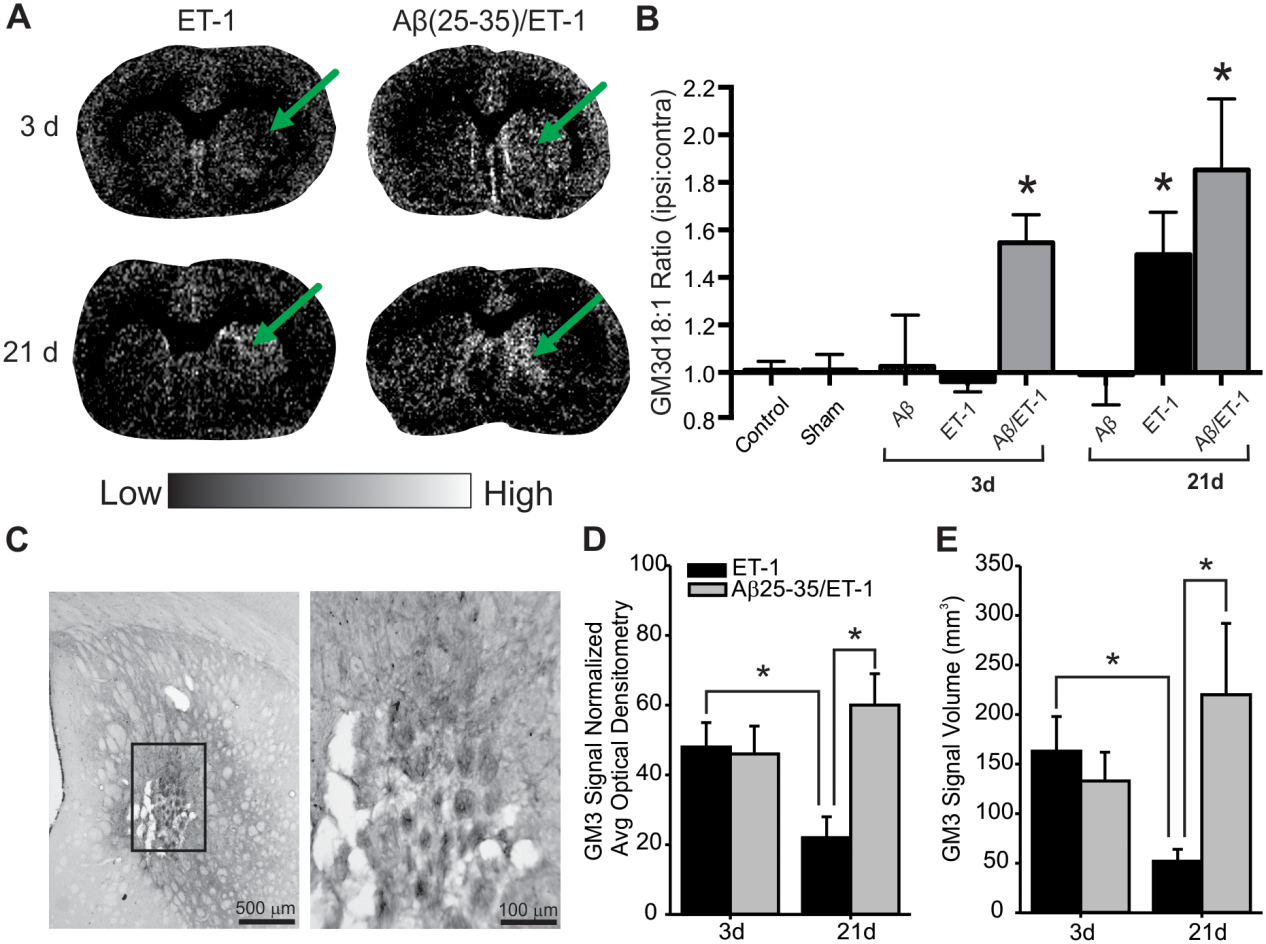
Increased GM3 Expression Within Infarcted Region of Combined Aβ/ET-1 Group Persists at 21 d. (A) Representative MALDI IMS images of ganglioside GM3 d18:1 in stroke (ET-1 alone) and combined Aβ/ET-1 animals 3 and 21 d following surgery. Arrows indicate region of stroke induced infarct. Light regions in images represent areas of high expression while dark regions represent low levels of expression as indicated by intensity bar. (B) MALDI IMS quantification of ROIs from the striatum of control, sham surgery, Aβ alone, ET-1 alone, and combined Aβ/ET-1 animals at 3 d and Aβ alone, ET-1 alone, and combined Aβ/ET-1 animals at 21 d. Data are expressed as the ratio of ipsilateral to contralateral ROIs. * indicates statistical significance over control and sham surgical groups, one-way ANOVA, Tukey’s post-hoc, p<0.05 (n = 4 for each group). There was an increase in expression observed in both the ET-1 alone and combined Aβ/ET-1 groups from 3 to 21 d, however this was only statistically significant in the ET-1 alone group as the expression at 3 d was at control levels. (C,D) IHC detection and quantification of GM3 within the infarct region. Panel (D) is a higher magnification image of panel (C). * indicates statistical significance between groups, one-way ANOVA, Tukey’s post-hoc, p<0.05 (n = 6 for each group).

MALDI IMS results indicated a persistent perturbation in GM3 expression following stroke within the stroke region. Even though the AB Sciex 4700 MALDI instrument was capable of differentiating between the d18:1 and d20:1 species of GM3, signals from the d20:1 species fell below the threshold of detection and only signals from the d18:1 species were detected and quantified. At 3 d following surgery, MALDI IMS analysis demonstrated that GM3 expression did not change following ET-1 injection alone or Aβ injections alone, however did increase in the combined Aβ/ET-1 group (Fig 3A and 3B) within the stroke region. By 21 d after surgery, GM3 expression remained at control levels in the Aβ alone group while GM3 significantly increased in the ET-1 alone group and remained elevated in the combined Aβ/ET-1 group within the stroke region (Fig 3A and 3B). To complement the MALDI IMS data, GM3 was detected by IHC 3 and 21 d following surgery. There was no GM3 signal detected in control treated animals (data not shown). By 3 d following surgery, GM3 signal was detected within the stroke region of both ET-1 alone and combined Aβ/ET-1 groups (Fig 3C and 3D). By 21 d following surgery, GM3 expression decreased within the infarct region of the ET-1 alone group compared to 3 d levels, whereas GM3 expression increased in the combined Aβ/ET-1 group over 3 d levels (Fig 3C and 3D). Similar to control and sham treated rats, there were no differences in GM3 expression (and all other gangliosides evaluated) within the right and left striatum of Aβ alone rats.

**Fig 4:**
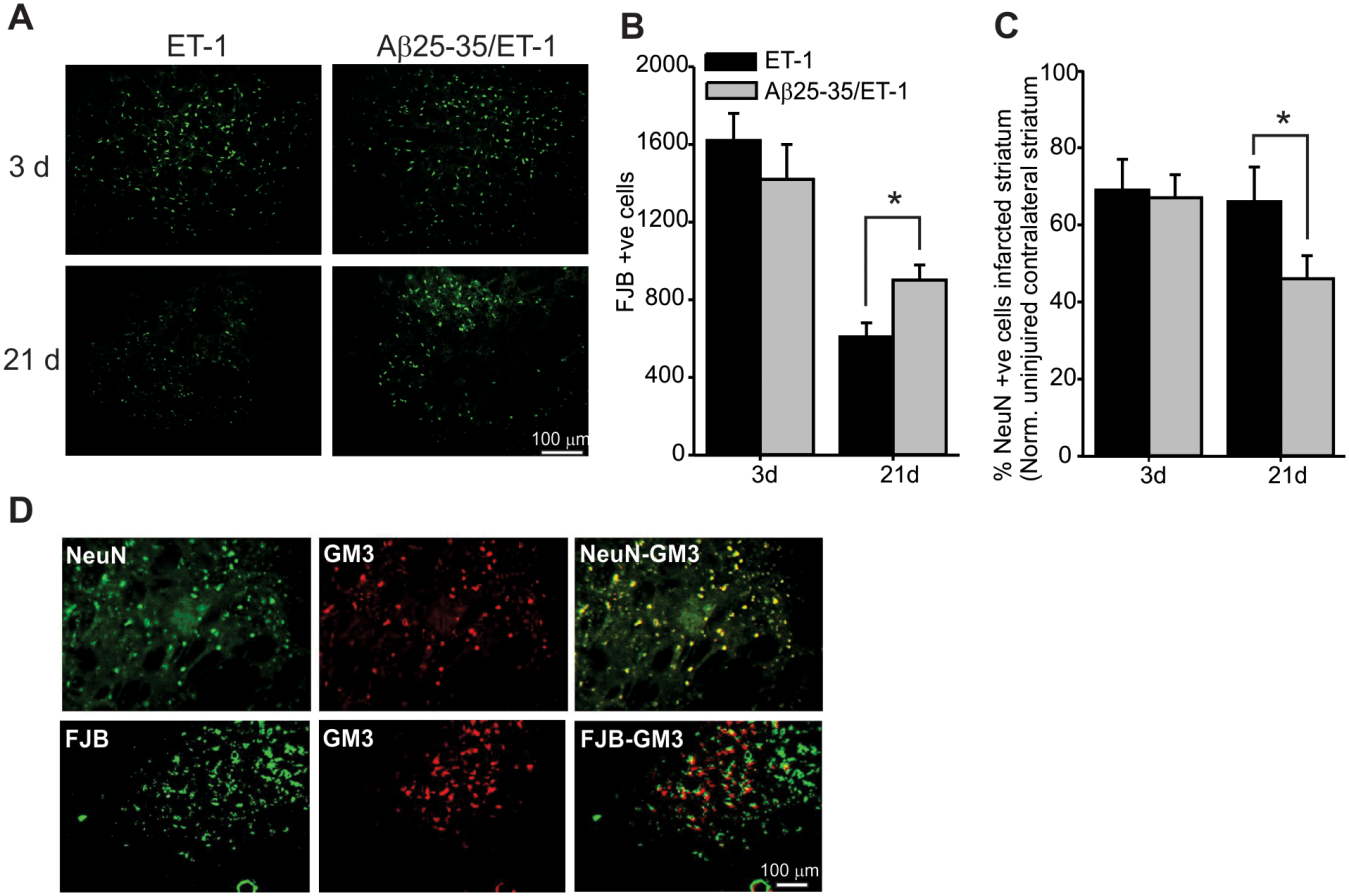
Effects of GM3 Accumulation on Neurodegeneration Following Stroke. (A) Photomicrographs of fluorojade-B immunofluorescence staining within the infarcted striatum of ET-1 and combined Aβ/ET-1 coronal rat brain sections. (B and C) Quantification of cell counts of FJB and NeuN cell counts. At 21 d following surgery, there was more FJB positive cells and less NeuN positive cells in the combined Aβ/ET-1 group compared to the ET-1 group according to one-way ANOVA and Tukey’s post-hoc, p<0.05 (n = 6 for each group). (D) Photomicrographs of immunofluorescence dual labelling showing co-localization between NeuN and GM3 (shown individually and overlayed) and FJB and GM3 (shown individually and overlayed) in the infarct region of a 21 d combined Aβ/ET-1 animal.

To evaluate the consequence of elevated GM3 expression within the stroke region, levels of cellular degeneration and neuronal loss were assessed. FluoroJade B (FJB) staining, a non-specific marker of cell death, was used to assess cellular degeneration at both 3 and 21 d after surgery. Extensive FJB positive staining was observed within the borders of the infarct in both the ET-1 alone and combined Aβ/ET-1 groups (Fig 4A). Quantification of FJB positive cells revealed no significant difference between the ET-1 alone and combined Aβ/ET-1 groups at 3 d following surgery (Fig 4B). At 21 d post-surgery, the number of FJB-positive cells were lower in both ET-1 alone and combined Aβ/ET-1 groups compared to 3 d, however the combined group had significantly more degenerating cells (35% reduction from 3 d levels) within the ischemic region compared to the ET-1 alone group (65% reduction from 3 d levels). NeuN, a marker for mature neurons was used to assess the number of neurons that survived between 3 and 21 d within the infarcted striatum. At 3 d following stroke, there were equal numbers of NeuN positive neurons between ET-1 alone and combined Aβ/ET-1 groups. However, by 21 d, there were fewer NeuN positive neurons within the infarcted striatum in the combined group compared to the ET-1 alone group. Taken together with the FJB results, this suggests that the combined Aβ/ET-1 group experienced greater neuronal degeneration within the damaged striatum following stroke. To assess if GM3 expression was localized within neurons and if these neurons underwent degeneration, dual immunofluorescence labeling was performed (Fig 4D). There was a high level of co-localization between NeuN and GM3 as well as between FJB and GM3, indicating that GM3 was expressed in degenerating neurons following stroke.

**Fig 5:**
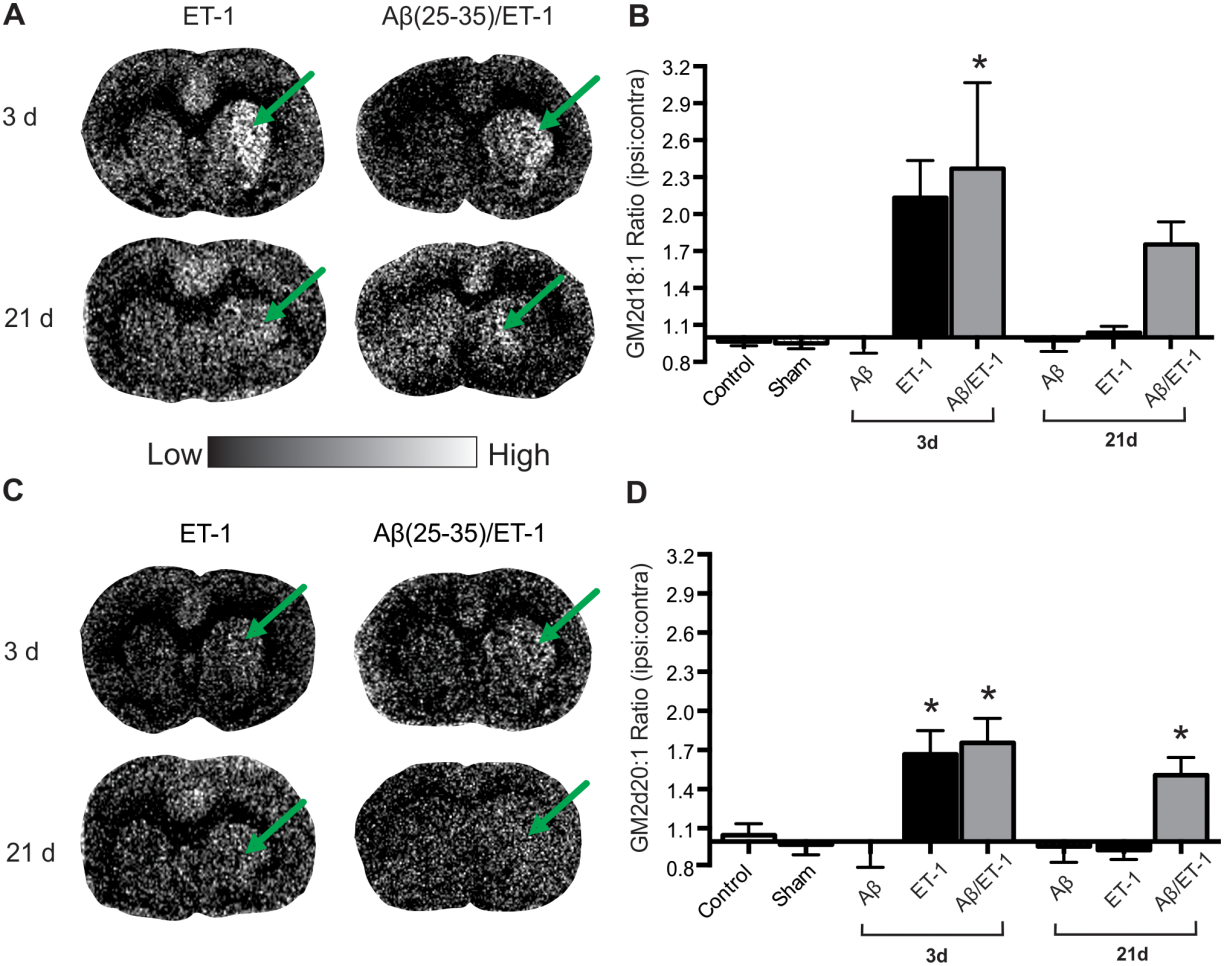
Elevated GM2 Expression at 3 d Remains Increased only in Combined Aβ/ET-1 Group. (A and C) Representative MALDI IMS images of GM2 d18:1 (A) and d20:1 (C) in stroke (ET-1 alone) and combined Aβ/ET-1 animals 3 and 21 d following surgery. Arrows indicate region of stroke induced infarct. (B and D) MALDI IMS quantification of ROI’s from the striatum of control, sham surgery, Aβ alone, ET-1 alone, and combined Aβ/ET-1 animals at 3 d and Aβ alone, ET-1 alone, and combined Aβ/ET-1 animals at 21 d. Data are expressed as the ratio of ipsilateral to contralateral ROIs. Light regions in images represent areas of high expression while dark regions represent low levels of expression as indicated by intensity bar. There was no statistical increase in GM3 expression between 3 and 21 d in any group, however, GM2 expression in the ET-1 group decreased significantly from 3 to 21 d (back to control levels). * indicates statistical significance over control and sham surgical groups, one-way ANOVA, Tukey’s post-hoc, p<0.05 (n = 4 for each group).

Since GM3 levels were differently expressed between surgical groups, we next evaluated the levels of GM2. Interestingly, GM2 expression levels were slightly different than the observed changes in GM3. The expression patterns of both the d18:1 and d20:1 species were profiled and quantified by MALDI IMS (Fig 5). By 3 d following surgery, both GM2 d18:1 and GM2 d20:1 expression in the combined Aβ/stroke group was found to be significantly elevated compared to Aβ alone and control groups (grey bars in Fig 5B and 5D). Although GM2 expression in the ET-1 alone group also showed a very strong trend of increased expression in the GM2 d18:1 species, it was not found to be statistically significant (black bars in Fig 5B). It was, however, found to be significantly elevated in the GM2 d20:1 species compared to Aβ alone and control groups (black bars in Fig 5D). At 21 d post-surgery, GM2 expression returned to control levels in the ET-1 alone group for both the d18:1 and d20:1 species, but remained increased in the combined Aβ/ET-1 group compared to Aβ alone and control groups (grey bars in Fig 5D), though this increased expression was only statistically significant in the d20:1 species.

**Fig 6:**
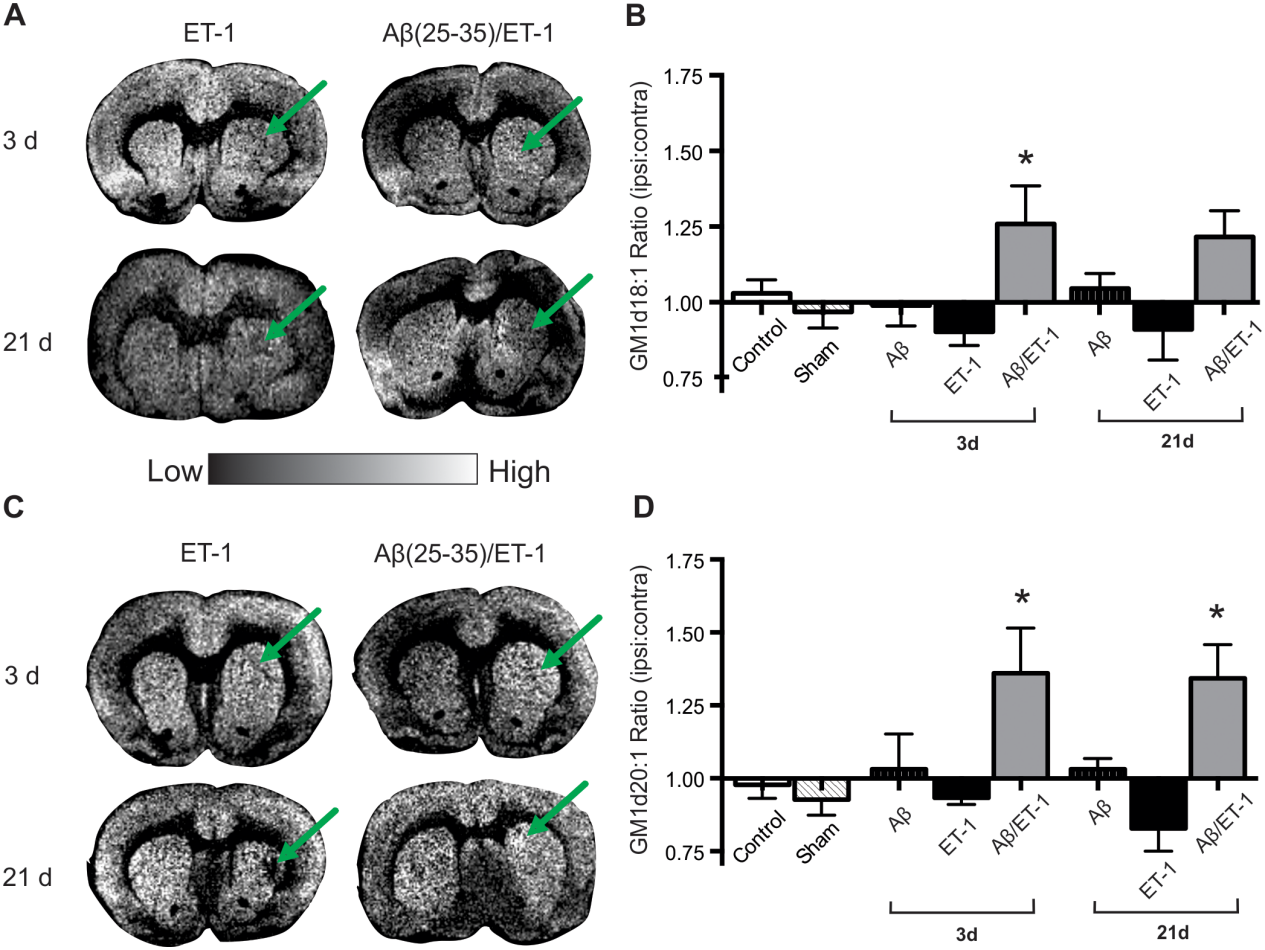
Elevated GM1 Expression in Combined Aβ/ET-1 Group. (A and C) Representative MALDI IMS images of GM1 d18:1 (A) and d20:1 (C) in stroke (ET-1 alone) and combined Aβ/ET-1 animals 3 and 21 d following surgery. Arrows indicate region of stroke induced infarct. (B and D) MALDI IMS quantification of ROI’s from the striatum of control, sham surgery, Aβ alone, ET-1 alone, and combined Aβ/ET-1 animals at 3 d and Aβ alone, ET-1 alone, and combined Aβ/ET-1 animals at 21 d. Data are expressed as the ratio of ipsilateral to contralateral ROIs. Light regions in images represent areas of high expression while dark regions represent low levels of expression as indicated by intensity bar. There were no statistically significant changes in GM1 expression between 3 and 21 d in any of the groups. * indicates statistical significance over control and sham surgical groups in panel (B) and statistical significance over control, sham and ET-1 alone surgical groups in panel (D), one-way ANOVA, Tukey’s post-hoc, p<0.05 (n = 4 for each group).

Analysis of GM1 d18:1 expression was performed and data indicated no statistical difference between the ET-1 alone, Aβ alone, and control groups 3 d following surgery, however, GM1 expression in the combined Aβ/ET-1 group was significantly elevated compared to the other surgical and control groups (Fig 6B). At 21 d, GM1 d18:1 expression showed the same expression profile between experimental groups with no statistical differences (Fig 6B). Analysis of the d20:1 species of GM1 demonstrated significantly increased expression in the combined Aβ/ET-1 group compared to all surgical and control groups at both 3 and 21 d post-surgery (Fig 6D).

**Fig 7:**
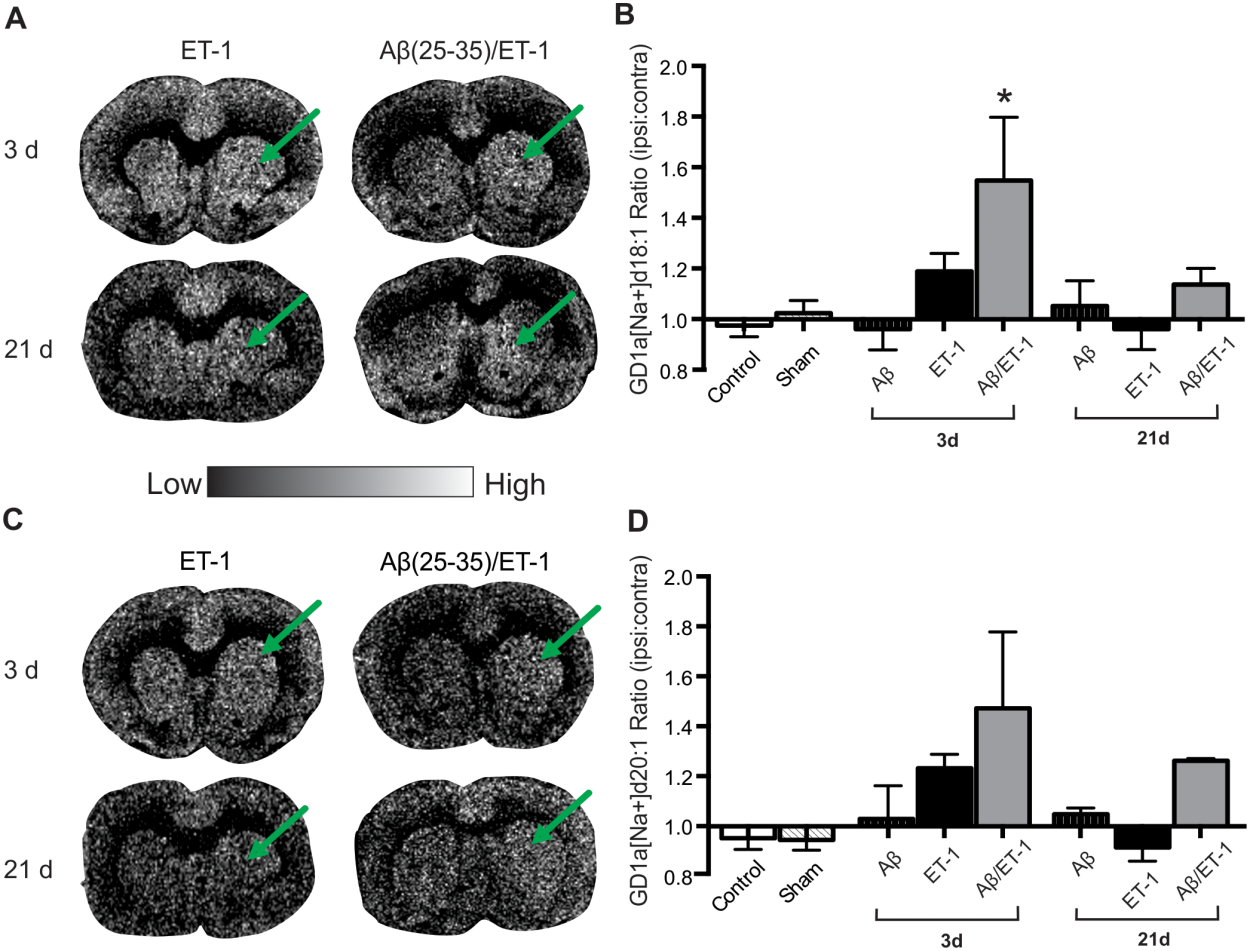
Increased GD1a [Na+] Expression at 3 d in Combined Aβ/ET-1 Group. (A and C) Representative MALDI IMS images of GD1a [Na+] d18:1 (A) and d20:1 (C) in stroke (ET-1 alone) and combined Aβ/ET-1 animals 3 and 21 d following surgery. Arrows indicate regions of stroke induced infarct. (B and D) MALDI IMS quantification of ROI’s from the striatum of control, sham surgery, Aβ alone, ET-1 alone, and combined Aβ/ET-1 animals at 3 d and Aβ alone, ET-1 alone, and combined Aβ/ET-1 animals at 21 d. Data are expressed as the ratio of ipsilateral to contralateral ROIs. Light regions in images represent areas of high expression while dark regions represent low levels of expression as indicated by intensity bar. GD1a [Na+] expression significantly decreased from 3 to 21 d in the d18:1 species. There were no other significant changes in expression between 3 and 21 d in any of the groups. * indicates statistical significance over control and sham surgical groups one-way ANOVA, Tukey’s post-hoc, p<0.05 (n = 4 for each group).

**Fig 8:**
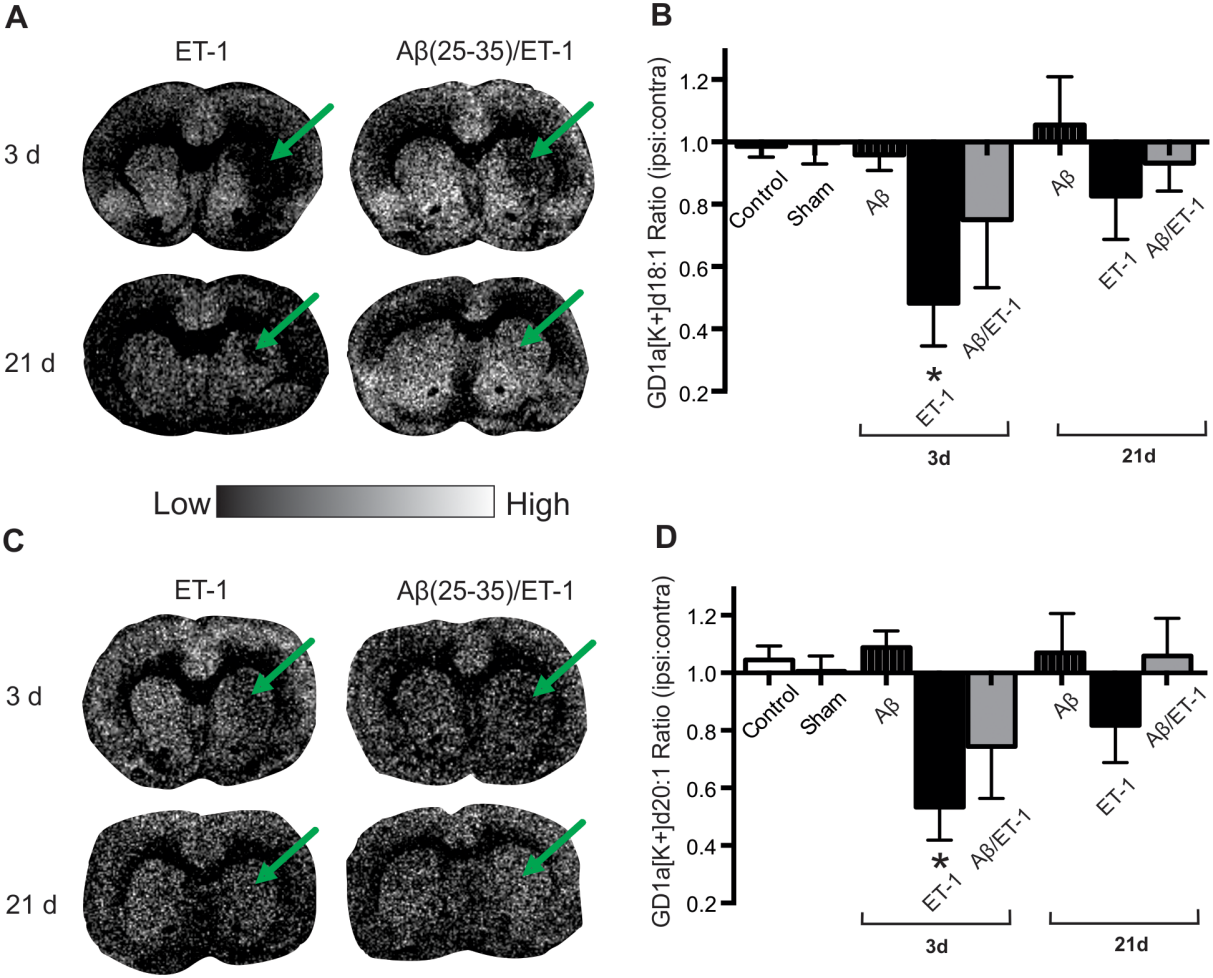
Decreased Expression of GD1a [K+] at 3 d in ET-1 Alone Group. (A and C) Representative MALDI IMS images of GD1a [K+] d18:1 (A) and d20:1 (C) in stroke (ET-1 alone) and combined Aβ/ET-1 animals 3 and 21 d following surgery. Arrows indicate regions of stroke induced infarct. (B and D) MALDI IMS quantification of ROI’s from the striatum of control, sham surgery, Aβ alone, ET-1 alone, and combined Aβ/ET-1 animals at 3 d and Aβ alone, ET-1 alone, and combined Aβ/ET-1 animals at 21 d. Data are expressed as the ratio of ipsilateral to contralateral ROIs. Light regions in images represent areas of high expression while dark regions represent low levels of expression as indicated by intensity bar. There were no statistically significant changes in expression between 3 and 21 d in any of the surgical groups. * indicates statistical significance over control groups, one-way ANOVA, Tukey’s post-hoc, p<0.05 (n = 4 for each group).

All MALDI IMS experiments presented in this work were performed using negative ion mode. The most intense signals observed for GM1, GM2, and GM3 were their singly charged anions, [M - H^+^]^1-^ (M minus H^+^ ions). Given that sialic acid is the most acidic site of these gangliosides, and each GM1, GM2, and GM3 has one sialic acid, the deprotonation site is likely the sialic acid. GD1a on the other hand is the only ganglioside studied in this work having two sialic acid groups. However, the doubly charged GD1a anion, [M - 2H^+^]^2-^, was not observed by MALDI MS. Instead, the sodium and potassium adducts were observed as two distinct peaks, [M - 2H^+^ + Na^+^]^1-^ and [M - 2H^+^ + K^+^]^1-^, likely due to its considerable presence in tissue. Interestingly, GD1a showed very different patterns of expression for the sodium and potassium adduct ions. (Figs 7 and 8). At 3 d post-surgery, GD1a [Na+] d18:1 showed significantly increased expression in the combined Aβ/ET-1 group compared to controls (Fig 7B and 7D). The d18:1 species expression in the combined Aβ/ET-1 group decreased at 21 d and was no longer different from the other groups.

The potassium adduct of GD1a, (GD1a [K+]) (Fig 8) showed a completely different pattern of expression from the GD1a [Na+]. At 3 d post-surgery, the ET-1 alone group demonstrated a significantly decreased expression of GD1a [K+] compared to Aβ alone and control groups, but this returned to control levels by 21 d, whereas no statistical difference was observed at either time point in the combined Aβ/ET-1 group, although there was a trend of decreased expression as well (Fig 8A and 8B). A similar trend was observed for the GD1a [K+] 20:1 species (Fig 8C and 8D).

## Discussion

Based on evidence from the literature [4,5,7,9,14–16,18,19], the observed increase in simple gangliosides GM2 and GM3 in the stroke region of the Aβ/ET-1 combined group at both 3 and 21 d after surgery is indicative of a more severe pathological response compared to ET-1 alone and Aβ alone as GM2 has been shown to be elevated immediately after TBI [14] and accumulations of GM3 has been shown to increase toxicity and induce apoptosis in cells [15–19]. The observed reduction of GM2 expression back to control levels following ET-1 alone at 21 d may imply that the brain has begun its repair process or the injury was less severe. GM2 expression levels within the combined Aβ/ET-1 rat brain remained elevated at 21 d which may indicate that the injury is persisting and thus more brain damage is occurring. This hypothesis supports previous pathological and behavioural findings using this combined animal model of Aβ/ET-1 in our lab [13,28,29,31,34]. It is possible that the observed accumulation of simple ganglioside species is a result of the breakdown of the more complex A-series gangliosides via enzymatic degradation; however, the expression patterns of both GM1 and GD1a observed in this study suggest that the degradation pathways may be more complex. A slight decrease in GM1 ganglioside occurred in the ET-1 alone group while an increase the simple species GM2 (at 3 d) and GM3 (at 21 d) was observed. This finding supports our initial A-series degradation hypothesis. Contrarily, in the combined Aβ/ET-1 group, in which previous studies has shown that stroke induced damage was more severe^27^, a significant increase in GM1 expression was observed despite there also being a significant increase in both GM2 and GM3 at both 3 and 21 d. This finding does not rule out the A-series degradation hypothesis but suggests that the mechanism of simple ganglioside accumulation is more complex than degradation along the A-series pathway. Ganglioside synthesis from the common precursor GM3 can occur by adding sugar units to the oligosacharride chain (i.e. along most of the A-series pathway) or by the addition of sialic acid residues (i.e. A to B-series pathway). It is possible that in the combined Aβ/ET-1 group, where an increase in both simple and complex A series ganglioside species was observed, the accumulation was due to the degradation of the sialic acid residues of B- or C-series gangliosides thus resulting in an increase in the A-series species. Further study into the enzymatic activities that regulate ganglioside metabolism during and post-brain injury is warranted to address this question.

The accumulation of the simple ganglioside species GM3 is of particular interest due to evidence supporting its potential apoptotic properties [15–19]. The immediate and significant increase along with the sustained expression of GM3 over time observed within in the combined Aβ/ET-1 rat brain may be reflective of the increased pathological response that was previously observed in this animal model [13,28,29,31,34]. Interestingly, GM3 did not show the same pattern of expression in the ET-1 alone brain, with a trend of increased expression only at 21 d post-surgery, suggesting that the interaction between Aβ and the stroke injury was responsible for the observed accumulation of GM3 in the combined group and may have played a mechanistic role in the synergism of these two pathologies. Unexpectedly, results from the GM3 IHC and MALDI IMS images from the ET-1 alone group (Fig 3) were not fully congruent. This may be explained by the difference in the techniques themselves as IHC and MALDI IMS do not necessarily measure the same thing, especially with respect to detection of lipids. IHC measures the ability of the ganglioside antibody to bind to, and label GM3 on cellular membranes while MALDI IMS measures the signal intensity of ions of a particular mass-to-charge ratio corresponding to GM3 molecules. The lack of specificity for ganglioside antibodies to bind and label tissues in IHC is a significant challenge in the glycosphingolipid field and thus is the principle reason MALDI IMS was chosen as the main technique in this study.

Previous work using a mouse model of stoke-reperfusion injury examined ganglioside expression in a number of key anatomical regions for ischemic risk, including the striatum, and found that there was an increase in complex ganglioside species GM1 and GD1a immediately after stroke which peaked at 3 d after injury then returned to basal levels at later time-points^13^. This trend is similar to the results of the combined group in the current study. However, following stroke alone there was a decrease in GM1 and GD1a [K+] expression after injury. The differences in these results may be explained by the type and severity of injury the animals received. Whitehead et al. (2011) used a mouse model of middle cerebral artery occlusion (MCAO) that causes a severe, focal ischemic insult that damages a large region within the cerebral cortex, striatum, and hippocampus. This contrasts with the site specific striatal ET-1 ischemia model used in this study which was chosen as it best emulates the smaller, lacunar strokes seen in aging individuals who are at risk of developing dementia. The combination of Aβ-toxicity and stroke resulted in a more severe response than stroke alone, which may explain why the results of that surgical group better resemble the results of the severe MCAO stroke injury [13].

Since the d18:1 and d20:1 species are hypothesized to have unique functions within the cell [10], where possible, both species were quantified and analyzed. Although the main patterns of expression were similar, significant changes in expression were observed in the d20:1 species that did not reach statistical significance in the d18:1 species. This leads to the possibility that a greater change in expression was occurring in the d20:1 species than its more prevalent d18:1 species within the site of ischemic injury; however, further investigations are needed to follow up on this finding. The significance of this finding lies in the potential for future therapeutics targeting gangliosides as the d20:1 form may prove to be a more efficient therapeutic target than the d18:1 species.

Although MALDI IMS has proven to be an invaluable and reliable tool for the investigation of ganglioside expression, this study is not without limitations. The most limiting factor to the use of MALDI IMS in clinically-relevant research is that it is generally considered to be only a semi-quantitative technique. According to Stoekli et al. (2007) and Lietz et al. (2013), MALDI IMS images differ from each other due to three main factors which vary from image to image: tissue heterogeneity, sample preparation, and ion suppression effects. These variations make it difficult to quantitatively compare between MALDI IMS images without producing a significant amount of error [35,36]. However, this study was uniquely designed to circumvent all major sources of error that arise from these factors. Firstly, MALDI IMS images from this study were not compared to each other but instead were compared to themselves. By comparing the stroke-injured hemisphere with the contralateral non-injured hemisphere, variability due to sample preparation and matrix application was negligible. Secondly, because the two quantified regions of interest were from the same tissue section on mirrored regions of each hemisphere of the brain, it can be assumed that any tissue heterogeneity would be equivalent in both regions and thus not a significant source of error. Finally, ionization suppression effects, which are mass spectrometry signals other than the signal of interest that suppress the overall signal obtained, can also be assumed to be equivalent based on this method of quantification. Quantification of MALDI IMS images remains a controversial and heavily researched area of study, and while our method of quantification does not eliminate all sources of error, variability, the largest source of error, was significantly minimized.

This study was able to show, for the first time, that ganglioside expression was not only altered in stroke injury but is also differentially altered in a comorbid rat model of Aβ toxicity and stroke. Translating these animal findings to a potential clinical environment, changes in ganglioside expression may be indicative of a new mechanism of synergy and possible site of intervention for those at risk or currently suffering from AD and stroke comorbidities.
